# Dietary Antioxidant Supplementation Promotes Growth in Senegalese Sole Postlarvae

**DOI:** 10.3389/fphys.2020.580600

**Published:** 2020-11-12

**Authors:** Maria J. Xavier, Sofia Engrola, Luis E. C. Conceição, Manuel Manchado, Carlos Carballo, Renata Gonçalves, Rita Colen, Vera Figueiredo, Luisa M. P. Valente

**Affiliations:** ^1^Centro Interdisciplinar de Investigação Marinha e Ambiental, Universidade do Porto, Matosinhos, Portugal; ^2^Instituto de Ciências Biomédicas Abel Salazar, Universidade do Porto, Porto, Portugal; ^3^Centro de Ciências do Mar, Universidade do Algarve, Faro, Portugal; ^4^SPAROS Lda., Olhão, Portugal; ^5^IFAPA Centro El Toruño, El Puerto de Santa Maria, Cádiz, Spain

**Keywords:** dietary antioxidants, muscle growth, myogenesis, protein degradation, Senegalese sole

## Abstract

Somatic growth is a balance between protein synthesis and degradation, and it is largely influenced by nutritional clues. Antioxidants levels play a key role in protein turnover by reducing the oxidative damage in the skeletal muscle, and hence promoting growth performance in the long-term. In the present study, Senegalese sole postlarvae (45 days after hatching, DAH) were fed with three experimental diets, a control (CTRL) and two supplemented with natural antioxidants: curcumin (CC) and grape seed (GS). Trial spanned for 25 days and growth performance, muscle cellularity and the expression of muscle growth related genes were assessed at the end of the experiment (70 DAH). The diets CC and GS significantly improved growth performance of fish compared to the CTRL diet. This enhanced growth was associated with larger muscle cross sectional area, with fish fed CC being significantly different from those fed the CTRL. Sole fed the CC diet had the highest number of muscle fibers, indicating that this diet promoted muscle hyperplastic growth. Although the mean fiber diameter did not differ significantly amongst treatments, the proportion of large-sized fibers (>25 μm) was also higher in fish fed the CC diet suggesting increased hypertrophic growth. Such differences in the phenotype were associated with a significant up-regulation of the *myogenic differentiation 2* (*myod2*) and the *myomaker* (*mymk*) transcripts involved in myocyte differentiation and fusion, respectively, during larval development. The inclusion of grape seed extract (GS diet) resulted in a significant increase in the expression of *myostatin1*. These results demonstrate that both diets (CC and GS) can positively modulate muscle development and promote growth in sole postlarvae. This effect is more prominent in CC fed fish, where increased hyperplastic and hypertrophic growth of the muscle was associated with an upregulation of *myod2* and *mymk* genes.

## Introduction

Aquaculture is the fastest-growing animal industry and one of the main sources of protein for human consumption (FAO, 2020). To achieve a sustainable and competitive industry, it is essential to investigate factors that control fish somatic growth (Dobly et al., 2004). Skeletal muscle is the edible part of the fish, and is mainly composed of myofibrils accounting for about two-thirds of the muscle protein mass (Mommsen, 2001). Unlike other vertebrates, fish species tend to grow indeterminately, and growth is the result of skeletal muscle accretion both by cellular hyperplasia and hypertrophy (Valente et al., 2013; Canada et al., 2019). Muscle formation, known as myogenesis, is a complex and highly organized process that comprises the recruitment of stem cells to a lineage of myogenic progenitor cells (MPCs), myoblast proliferation, cell cycle withdrawal, differentiation and fusion of myoblasts, and the maturation of muscle fibers (Johnston et al., 2011; Valente et al., 2013). Four muscle-specific basic helix-loop-helix transcription factors, called myogenic regulatory factors (MRFs), which include myoblast determination factor (*myod)*, myogenic factor 5 (*myf5*), myogenin (*myog*) and myogenic regulator factor 4 (*mrf4*), are key regulators of myogenesis (Rescan, 2001). The primary MRFs, *myod* and *myf5*, are essential for the commitment of mesodermal cells to myogenic fate (Rudnicki et al., 1993) whereas the secondary MFRs, *myog* and *mrf4*, participate in the differentiation of myoblasts into multinucleated myotubes (Rescan, 2001). Other factors controlling myogenesis are the myostatin (*msnt1*), that prevents the progression of myogenic cells into the cell division cycle (Rescan, 2005) and the *myomarker (mimk*) that regulates fusion of these cells to form the multinucleated muscle fibers (Landemaine et al., 2014; Huang et al., 2019).

Skeletal muscle homeostasis relays on other key elements essential for structural and recycling pathways. The myosin heavy chain (*myhc*) is the major structural protein of the muscle (Hevrøy et al., 2006; Zhang et al., 2019). However, muscle mass is dependent on the balance between protein synthesis and concurrent protein degradation that in turn are key factors to regulate growth rates and protein retention (Conceição et al., 2001; Fraser and Rogers, 2007; Canada et al., 2019). The ubiquitin-proteasome pathway is responsible for the majority of protein degradation clearing damaged or aged proteins and removing of molecules endowed with regulatory functions (Collins and Goldberg, 2017). Muscle-specific ubiquitin ligases such as *murf1* and *mafbx* increase in the skeletal muscle during atrophy process (Fearon et al., 2012; Bower et al., 2009). This balance between protein synthesis and degradation is dynamic and largely influenced by intrinsic factors like genotype, age or sex (Peragon et al., 2001; Cleveland and Weber, 2011), and extrinsic such as temperature (Vieira et al., 2012; Campos et al., 2013a,c), photoperiod (Giannetto et al., 2013; Navarro-Guillen et al., 2018; Hou et al., 2019) and nutrition (Efeyan et al., 2015; Rocha et al., 2015, 2016; Canada et al., 2018, 2019).

Nutrition is generally recognized as a key factor controlling fish growth and health status. This is of particular interest in young fish in which functional microdiets that contain essential nutrients supplemented with nutraceuticals can enhance the production of high-quality juveniles (Gatlin, 2002). Several plant-derived extracts containing a wide spectrum of bioactive molecules that act as appetite enhancers, growth promoters and immunostimulants have been reported in finfish species (Reverter et al., 2014). The grapes of common grapevine (*Vitis vinifera*) are consumed since the ancient times for their nutritional and medicinal values. A grape seed extract contains about 5–8% polyphenols, including several flavonoids (e.g., catechin and epicatechin), procyanidins and phenolic acids (El-Beshbishy et al., 2009; Bijak et al., 2012). Such an extract has demonstrated a potent antioxidant effect in skeletal muscle both *in vitro* and *in vivo* (Vasilaki and Jackson, 2013; Wang et al., 2014; Haramizu et al., 2017). Another substance with powerful anti-inflammatory and antioxidant properties is curcumin, a yellow pigment extracted from the rhizome of turmeric (*Curcuma longa*) (Abrahams et al., 2019) that can also modulate muscle protein degradation (Busquets et al., 2001; Lin, 2007; Alamdari et al., 2009). Due to their activity as reactive oxygen species (ROS) scavengers, these bioactive molecules are of high interest as dietary supplements for fish species, with particular relevance during early developmental stages where growth potential is largely affected by nutritional clues. Nutritional events occurring during critical windows of development, may have long-term consequences on somatic structures, physiological functions or metabolic status of the organism (Waterland and Jirtle, 2004). The perspective of applying this novel concept of nutritional programming to fish nutrition provides numerous possibilities mainly focused on tailoring specific metabolic pathways or functions in farmed fish species (Geurden et al., 2013; Rocha et al., 2016; Canada et al., 2018) in order to identify best dietary formulations able to promote growth.

The aim of this study was the evaluation of dietary natural extracts (curcumin and grape seeds) as growth modulators in *Solea senegalensis* postlarvae. Growth, muscle cellularity and muscle development/growth expression patterns were established, and a possible epigenetic regulation was addressed. Analysis of dietary effects on sole antioxidant system will be presented in a companion manuscript (Xavier et al., 2021).

## Materials and Methods

### Experimental Diets

Three diets were tested in this study, including a commercial diet (WINFlat, SPAROS Lda., Portugal) used as the control (CTRL diet). This diet contains ingredients such as krill meal, squid meal, wheat gluten, fish meal, shrimp meal, fish hydrolyzate, pea protein concentrate, fish gelatin, fish oil, lecithin and a micronutrient premix comprising vitamins, minerals and other additives. Moreover, two experimental diets were prepared by supplementing the CTRL diet with an antioxidant extract of either curcumin (CC diet) at 46 g/kg of the micronutrient premix, or grape seed (GS diet) at 12 g/kg of the micronutrient premix. These selected doses of each antioxidant extract are under a patent pending application (PCT/IB2020/056001), and were chosen based on preliminary trials conducted at CCMAR (unpublished data). Curcumin (diferuloylmethane), the primary bioactive substance in turmeric, had a 95.34% purity, whilst the grape seed extract had 80% polyphenols, 30% procyanidolic polymers, and 12% oligomeric proanthocyanidins (OPCs). All diets were prepared by SPAROS Lda. (Olhão, Portugal). Feed samples were freeze-dried, ground and analyzed for dry matter (105°C for 24 h), crude protein by automatic flash combustion (Leco FP-528, Leco, St. Joseph, United States; N × 6.25), lipid content by petroleum ether extraction using a Soxtherm Multistat/SX PC (Gerhardt, Königswinter, Germany; 150°C), and gross energy in an adiabatic bomb calorimeter (Werke C2000; IKA, Staufen, Germany). Diet proximal information about diets is indicated in Table 1.

**TABLE 1:** Proximate analyses of the experimental diets.

Proximate analyses (% dry matter)	Diets		
	
	CTRL	CC	GS
Crude protein (% DM)	65.0	65.3	65.8
Crude fat (% DM)	19.4	19.9	19.5
Gross energy (MJ kg^–1^)	22.9	22.5	22.9

### Husbandry and Experimental Set-Up

This experiment was carried out by trained scientists and followed the European Directive 2010/63/EU of European Parliament and of the Council of European Union on the protection of animals used for scientific purposes and was approved by the Committee of Ethic and Animal Experimentation of the Centre of Marine Sciences of Algarve (CCMAR). The CCMAR (Faro, Portugal) facilities and their staff are certified to house and conduct experiments with live animals (‘group-1’ license by the ‘Direcçãaþo Geral de Veterinaria’, Ministry of Agriculture, Rural Development and Fisheries of Portugal).

Senegalese sole postlarvae originating from IPMA-EPPO (Olhão, Portugal) were transferred to a recirculation aquaculture system at CCMAR facilities, and acclimatized for 1 week prior to the feeding experiment. The Senegalese sole postlarvae were reared for 25 days, from 45 days after-hatching (DAH) to 70 DAH, under optimal environmental and zootechnical conditions. Postlarvae were kept in flat-bottom tanks (30 × 70 × 10 cm; 21 L), each tank stocking 630 individuals (3,000 ind/m^2^). The dietary treatments (CTRL, CC and GS diets) were randomly assigned to replicate tanks (*n* = 3 tanks per treatment). The system was equipped with a mechanical filter, a submerged and a trickling biological filter, a protein skimmer (AB Aqua Medic GmbH, Bissendorf, Germany) and UV sterilizer (Tropical Marine Centre Ltd, Hertfordshire, United Kingdom). Abiotic parameters were measured, and mortality was recorded daily; dead postlarvae were removed and the rearing units were carefully cleaned with minimal disturbance. Dissolved oxygen in water was 96.6 ± 7.2% of saturation, temperature was 19.6 ± 0.5°C, salinity was 30.4 ± 0.7 ppt and ammonia and nitrites was <0.1 mg/L. A 10:14 h light/dark photoperiod cycle was maintained, and the light intensity was 400 lx at water surface. Inert diet was delivered semi-continuously with automatic feeders for 24 h (cycles of 2 h of feeding followed by 1 h break). The amount of feed distributed to each tank was based on predicted maximum growth and daily adjustments were done based on visual inspection to avoid a large excess of uneaten feed (Engrola et al., 2005).

### Growth Performance

At the beginning (45 DAH) and by the end of the experiment (70 DAH), 60 and 120 postlarvae, respectively, were killed by using an overdose of anesthetic 2-phenoxyethanol (1000 ppm; Prolabo, VWR International LLC, Radnor, United States) then individually sampled for dry weight (DW; mg) and body length [Standard length (SL) mm] determination. Individual SL was determined using Axio Vision L.E. 4.8.2.0 (Carl Zeiss Micro Imaging GmbH, Oberkochen, Germany) and DW was determined in freeze-dried postlarvae (0.001 mg precision). Survival rate (%) was calculated as the percentage of estimated postlarvae at the end of the trial relative to their initial number in each tank.

### Morphometry

Six postlarvae at the beginning and five at the end of the trial were photographed for SL determination and fixed in 4% paraformaldehyde (Sigma-Aldrich, St. Louis, United States) in phosphate buffered saline (PBS tablets, Sigma-Aldrich, St. Louis, United States). After 24 h the samples were washed in PBS and stored in ethanol 70% at 4°C. Postlarvae were then decalcified with 10% EDTA pH 7.4. Samples were dehydrated in a graded ethanol (AGA, Prior Velho, Portugal) series, cleared in xylol (Prolabo, VWR International LLC, Radnor, United States) and included in paraffin Histosec^®^ (Merck, Whitehouse Station, United States). Each larva was sectioned (7 μm) transversely to the body axis at the anal opening level, mounted on adhesive slides, and stained with hematoxylin-eosin (Merck, Whitehouse Station, United States) before placing a cover slip.

Morphometric variables were measured in transversal body sections of individual fish, at the level of the anal opening. The total cross-section area [CSA (mm^2^)] of the postlarvae and the total cross-section of the muscle [Muscle CSA (mm^2^)] were measured after tracing the physical limits of those sections on the monitor, at a 400x magnification. Six and 10 photos were taken from representative parts of the muscle cross-sectional area in postlarvae with 45 and 70 DAH, respectively, at a 400x magnification. The number of fast-twitch fibers (N) was recorded in each photo. Total number of fibers in the muscle CSA was estimated by extrapolation of the mean number of fibers per photo relatively to the total muscle cross-sectional area. The fiber density (total number of fibers/mm^2^) was calculated by dividing the total number of fibers (N) by the total cross section muscle area [Muscle CSA (mm^2^)]. The area (μm^2^) of a minimum of 900 fibers per cross-section was measured per fish following previous studies (Campos et al., 2013a). The fast-twitch fiber diameter (μm) was indirectly estimated using the fiber area and assuming that muscle fibers are round shaped as follows: d (μm) = 2√ (a (μm^2^)/π), where d is the diameter of fast twitch fibers and a is the fiber area. This morphometric study was performed using an Olympus BX51 microscope (Olympus Europa GmbH, Hamburg, Germany) with the Cell^B Basic imaging software and photos capture with CCD-video camera (ColorView Soft Imaging System, Olympus).

### RNA Isolation and RT-qPCR Analysis

For gene expression analysis, four individual postlarvae of each dietary treatment at the end of the growth trial were snap-frozen in liquid nitrogen and preserved in −80°C until use. For the RNA extraction, as postlarvae were too small to simply dissect muscle, the head and the caudal part of body were removed, and the squared region mainly containing muscle and viscera were selected for RNA-isolation.

Samples were homogenized using a Fast-prep FG120 instrument (Bio101 INC, Vista, United States) and Lysing Matrix D (Q- Bio- Gene, Irvine, United States) with 1 ml Tri Reagent (Sigma-Aldrich, St. Louis, United States) for 60 s at speed setting 6. Chloroform (0.2 ml) was added to each sample before centrifuging at 14.000 rpm for 15 min. The supernatant content was transferred to columns of the Isolate II RNA Mini Kit (Bioline, London, United Kingdom) and total RNA was treated twice for 30 min with DNase I following the manufacturer’s protocols. Total RNA quality was checked by agarose gel electrophoresis and a Nanodrop ND-8000 (Thermo Scientific, MA, United States) was used to determine its concentration. One μg of total RNA was reverse-transcribed using the iScript^TM^ cDNA Synthesis kit (Bio-Rad, Berkeley, United States) according to the manufacturer’s protocol.

The qPCR assays were performed in duplicate in a 10 μL volume containing cDNA generated from 10 ng of the original RNA template, 300 nM of each specific forward and reverse primers, and 10 μl of iQ^TM^ SYBR^®^ Green Supermix (Bio-Rad, Berkeley, United States). The genes analyzed involved in the regulation of muscle development and growth were the myogenic factor 5 (*myf5*), muscle-specific regulatory factor 4 (*mrf4*), myogenin (*myog*), myoblast determination protein 2 (*myod2*), myosin heavy chain (*myhc*), myostatin 1 (*mstn1*), myomarker (*mymk*), muscle ring-finger protein-1 (*murf1*) and muscle atrophy F-box (*mafbx*). Expression of DNA methyltransferases *dnmt1*, *dnmt3aa*, *dnmt3ba* and *dnmt3bb.1* were also analyzed. Species-specific primers for qPCR are indicated in Table 2. Primers for Senegalese sole *dnmt1*, *dnmt3aa, dnmt3bb.1* were previously published (Manchado et al., 2008; Firmino et al., 2017). The qPCR amplification protocol was as follows: 7 min for denaturation and enzyme activation at 95°C followed by 40 cycles of 30 s at 95°C and 1 min at 60°C. Expression data were normalized using the geometric mean of two reference genes, ubiquitin (*ubi*) and glyceraldehyde-3-phosphate dehydrogenase 2 (*gadph2*) (Infante et al., 2008) and the relative mRNA expression calculated using the comparative Ct method (Livak and Schmittgen, 2001).

**TABLE 2:** Primers used in qPCR.

Gene	Fwd sequence (5′ → 3′)	Rev sequence (5′ → 3′)	Accession nr (GenBank)	Size (bp)
*myf5*	CCGACGCCTGAAGAAAGTCAACCACGCT	CACCTTGGGCAGGCGCTGGCT	FJ515910	85
*myod2*	GTTGCACAGCCACCAGCCCAAACCAGC	TCCCGGCCAGTTCTCAGCAGTGCCT	FJ009108	106
*myog*	GCATGTCACCTCACTCCGAGCCCACT	GCAGCTCTCCGACGGTCCATGGTCACT	EU934044	103
*mrf4*	CACGGACAGACGCAAGGCCGCCAC	TGGTTGGGGTTGGCCACGGTCTTCCT	EU934042	105
*mymk*	GTTGCTGCTTCGGTGCCCTTGCTCTGAT	GCAGGATGAAGGACACAGCCAGAGACAGAT	unigene25840*	111
*myhc*	TGCCAAGTTGAGCAAAGAGAAGAAAGCCCT	TGTTCCAGCTTTGTCTTGGCCTTGGTGAGA	FJ515911	120
*mstn1*	GACGAGCACGCCACCACGGAGACGA	TGGCTGCCCGTTCACCTGCACCACT	EU934043	78
*murf1*	GCTGCCCCATCTGCCTGGAAATGTTCACC	AGGTCACTGGCACAACTACGGCAGAGG	unigene284737*	88
*marfbx*	TTCTTGATGACCAGCAGAACGTCCGTCCA	TGCCCATGTCCTGAACCAGACCGCACAG	unigene22801*	90

Gene name, sequences, Accession numbers at GenBank or SoleaDB and amplicon sizes are indicated. *Sequences available at SoleaDB (Benzekri et al., 2014).

### Statistical Analysis

All data were tested for normality using a Kolmogorov–Smirnov (whenever *n* > 30) or Shapiro–Wilk (whenever *n* < 30) test and for homogeneity of variance using a Levene’s test. Data were log transformed when required and percentages were arcsin transformed prior analysis. Comparisons between groups fed different diets were made using one-way ANOVA followed by a Tukey *post hoc* test for growth performance, morphometry and gene expression. A Pearson’s coefficient correlation was used to relate the relative expression of genes regulating muscle growth with muscle parameters. In all cases, significant levels were set at *P* < 0.05. All tests were carried out using IBM SPSS Statistics v19 software.

## Results

### Effects of Diets on Growth Performance

Postlarvae average initial DW at 45 DAH was 12.3 ± 5.7 mg. After rearing sole for 25 days, postlarvae fed with CC and GS diets had a higher DW and SL than the CTRL group (*P* < 0.001) (Table 3). The increase in DW was 17.3 and 11.7% higher in CC and GS fed fish, respectively. At the end of the experiment postlarvae survival rate was around 98% with no differences between dietary treatments.

**TABLE 3:** Growth performance and muscle cellularity parameters of Senegalese sole post-postlarvae at the beginning (45 DAH) and the end of the growth trial (70 DAH).

		Treatments			
		
	Initial	CTRL	CC	GS	*P*
DW (mg)	12.3 ± 5.7	163.8 ± 40.2^b^	192.2 ± 39.4^a^	182.9 ± 44.8^a^	<0.001
SL(mm)	17.7 ± 3.1	35.0 ± 2.9^c^	39.1 ± 2.7^a^	37.8 ± 2.8^b^	<0.001
CSA (mm^2^)	4.1 ± 0.6	13.4 ± 1.8^b^	17.2 ± 1.9^a^	15.1 ± 1.6^ab^	0.047
Muscle CSA (mm^2^)	1.0 ± 0.1	4.4 ± 0.6^b^	5.7 ± 0.7^a^	4.7 ± 0.1^ab^	0.015
Number of fibers (N)	5051.9 ± 749.4	17983.6 ± 1438.6^b^	21906.5 ± 1814.5^a^	20551.2 ± 2325.7^ab^	0.046
Fiber density (N/μm^2^)	5211.2 ± 695.1	4130.7 ± 289.7	3856.6 ± 388.0	4347.0 ± 555.0	0.280
Fibers diameter (μm)	12.8 ± 0.9	15.2 ± 0.6	16.1 ± 1.0	15.6 ± 1.4	0.809

*Values are means ± SD, *n* = 60 and 120 for sole growth performance at 45 DAH and 70 DAH, and *n* = 6 and 5 for muscle cellularity parameters at 45 DAH and 70 DAH, respectively. Different superscript letters indicate significant differences (*P* < 0.05, one-way ANOVA) between the dietary treatments at 70 DAH.*

### Effects of Diets on Muscle Development

**FIGURE 1:**
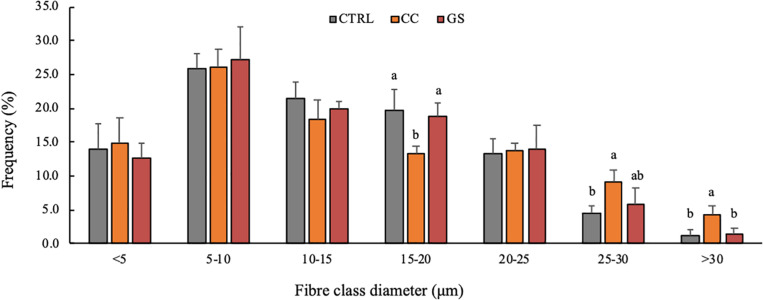
Frequency of fibers per class diameter in Senegalese sole, at 70 DAH. Values are presented means ± SD (*n* = 5). Different superscript letters indicate significant differences (*P* < 0.05, one-way ANOVA) between the dietary treatments (CTRL, CC and GS).

**FIGURE 2:**
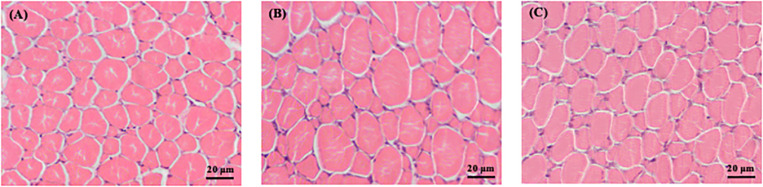
Transversal cross-section area of skeletal muscle fast-twitch fibers of Senegalese sole, at 70 DAH, of the different treatments **(A)** CTRL, **(B)** CC, and **(C)** GS. Magnification: 400x. Scale bars: 20 μm.

The enhanced growth observed in sole postlarvae fed CC and GS diets was associated with a higher CSA and muscle CSA (Table 3). However, these differences were only significant in postlarvae fed with the CC diet in relation to the CTRL treatment (*P* < 0.05). Similarly, fish fed CC and GS diets displayed a higher number of muscle fibers than the CTRL fed fish, but these differences were only statistically significant in the former [1.2-fold higher than the CTRL (*P* = 0.046)]. The fiber density and the mean fiber diameter of the postlarvae did not differ among treatments, however, the proportion of large-sized fibers (>25 μm) was significantly higher in postlarvae fed the CC diet than in all other dietary treatments (Figures 1, 2).

### Effects of Diets on Gene Expression Patterns

**FIGURE 3:**
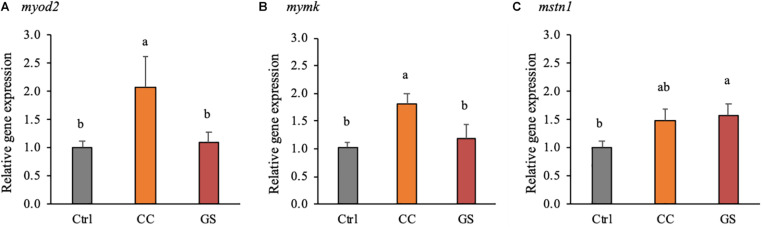
Expression of genes encoding for muscle development and epigenetic regulation: *myod2* **(A)**, *mymk* **(B)**, and *mstn1* **(C)**, at 70 DAH (*n* = 4). mRNA expression was normalized to transcript levels of ubq and rps4. Values are presented means ± SEM. Different superscript letters indicate significant differences (*P* < 0.05, one-way ANOVA) between the dietary treatments (CTRL, CC, and GS).

The expression levels of 13 genes related to muscle development and growth is depicted in Table 4. Dietary CC and GS can modulate the expression of *mymk*, *myod 2* and *mstn 1* in sole postlarvae. The *mymk* and *myod2* mRNA levels were significantly higher in postlarvae fed the CC diet than in those fed GS or CTRL diets (*P* < 0.05; Figures 3A,B). These two genes were positively correlated with both the large-sized fibers (>30 μm) and muscle CSA (Table 5). The expression of *mstn1* was up-regulated in fish fed diet GS compared to those fed the CTRL (*P* = 0.028; Figure 3C). No significant differences were observed in the expression of muscle-specific ubiquitin-proteasome genes among fish fed the experimental diets. In the case of DNA methyltransferases, a positive correlation between *dnmt1* and *dmnt3bb1* with fiber diameter and d*nmt3bb1* with large sized fibers (>30 μm) was observed, although the differences between dietary treatments were not significant.

**TABLE 4:** Expression of genes encoding for muscle development and epigenetic regulation: *myf5, myog, mrf4, myhc, murf1, mafbx, dnmt1, dnmt3aa, dnmt3ba*, and *dnmt3bb.1* at 70 DAH (*n* = 4).

	CTRL	CC	GS	*P*
*myf5*	1.1 ± 0.4	1.7 ± 0.6	1.7 ± 0.2	0.203
*myog*	1.1 ± 0.3	2.0 ± 0.6	1.4 ± 0.1	0.067
*mrf4*	1.0 ± 0.3	1.5 ± 0.3	1.4 ± 0.2	0.166
*myhc*	1.0 ± 0.1	1.3 ± 0.5	1.1 ± 0.2	0.610
*murf1*	1.1 ± 0.3	2.3 ± 0.7	2.1 ± 1.0	0.070
*mafbx*	1.1 ± 0.4	2.9 ± 2.7	1.0 ± 0.2	0.440
*dnmt1*	1.0 ± 0.3	1.6 ± 0.6	1.0 ± 0.2	0.154
*dnmt3aa*	1.0 ± 0.1	1.3 ± 0.5	1.0 ± 0.2	0.854
*dnmt3ba*	1.0 ± 0.3	1.0 ± 0.3	0.7 ± 0.1	0.378
*dnmt3bb.1*	1.0 ± 0.2	1.3 ± 0.3	0.9 ± 0.2	0.155

*Values are means ± SEM. Absense superscript letters indicate no differences between the dietary treatments (CTRL, CC and GS) (*P* > 0.05).*

**TABLE 5:** Correlations (Pearson’s coefficient, *n* = 3) between muscle cellularity, somatic growth or gene expression data in Senegalese sole post-postlarvae, at 70 DAH.

	*myod2*	*myog*	*mynk*	*mafbx*	*dnmt1*	*dnmt3bb.1*
Muscle CSA (mm^2^)	0.84*	n.s	0.82*	n.s	n.s	n.s
Total number of fiber (N)	n.s	n.s	n.s	n.s	n.s	n.s
Fiber Density (N/mm^2^)	n.s	n.s	n.s	n.s	n.s	−0.83*
Mean fiber diameter (μm)	n.s	n.s	n.s	n.s	0.90*	0.90**
Fiber Classes (μm):	n.s	n.s	n.s	n.s	n.s	n.s
<5	n.s	n.s	n.s	0.94**	n.s	n.s
5–10	n.s	n.s	n.s	n.s	n.s	n.s
10–15	n.s	n.s	n.s	n.s	n.s	n.s
15–20	n.s	−0.86*	−0.93**	n.s	n.s	n.s
20–25	n.s	n.s	n.s	n.s	n.s	n.s
25–30	n.s	0.87*	0.93**	n.s	0.87*	0.86*
>30	0.95*	n.s	0.88*	n.s	n.s	0.82*

*ns, non-significant at *P* > 0.05. ^∗^Correlation is significant at *P* < 0.01. ^∗∗^Correlation is significant at *P* < 0.05.*

## Discussion

Curcumin and grape seed extracts have been shown to have a wide pharmacological effect as antioxidant, anti-inflammatory, anti-carcinogenic and anti-bacterial (El-Beshbishy et al., 2009). In this study, we demonstrate a positive modulatory effect on muscle development and growth of curcumin and grape seed extracts, when added as dietary supplements in sole postlarvae. This growth-promoting effect of curcumin and grape seed is in accordance with previous studies in other fish species (Cui et al., 2013; Wilson et al., 2015; Jiang et al., 2016; Akdemir et al., 2017; Mahmoud et al., 2017; Kesbiç and Yigit, 2019). Both CC and GS diets significantly improved the DW and SL in relation to the CTRL fish. Fish from CC dietary treatment showed increased total number of muscle fibers that resulted in higher muscle CSA, suggesting that this supplement promotes hyperplastic growth in sole. The same trend is visible in the postlarvae from GT treatment, although without significant differences from the CTRL group. Although the mean fiber diameter was not significantly affected by the dietary treatments, a closer look into the fiber size distribution evidenced a significant increase in the proportion of the largest diameter fiber classes (> 25 μm) in fish fed the CC diet. This result together with increased number of total fibers explain the lack of significant differences in mean fiber diameter and fiber density between fish the CC and the CTRL diets. Therefore, curcumin supplementation seems to promote both hyperplasia and hypertrophy of muscle cells in postlarvae of sole.

Overall, in the present study, the performance of Senegalese sole was much better than that previously reported in literature at similar postlarvae stages. A DW between 30 and 102 mg was reported in postlarvae with 68 – 69 DAH (Engrola et al., 2005, 2009), or even fourfold lower values (age 67 – 74 DAH) (Morais et al., 2014; Pinto et al., 2016) than those presently observed (160–190 mg at 70 DAH). There are not many studies describing skeletal muscle growth in Senegalese sole at similar developmental stages. The closest study was carried out by Campos et al. (2013a) at 21°C and with sole fed a commercial diet: at 83 DAH, fish reached ∼80 mg DW and had a 3 mm^2^ muscle CSA comprising a total of ∼8000 fibers with a mean fiber diameter of ∼16 μm. In the present study, all postlarvae reached a higher body weight (increase of 2.2x) and larger muscle CSA (increase of 1.6x). This higher growth seems to be mainly achieved by a 2.5-fold increase in the total number of fibers, which is further supported by a much higher percentage (39%) of small-sized fibers (<10 μm) compared to that (11%) reported by Campos et al. (2013a). Moreover, although mean fiber diameter was similar in both studies, fibers also reached a larger maximum diameter (30 vs. 28 μm) in the present study. Therefore, differences in growth performance of sole seems to be explained, not only by increased hyperplastic growth, but also by hypertrophy. This high variability among studies for postlarvae DW, at similar age, is an indicator of how sole rearing protocols have improved in the last years. This is mainly due to a combination of rearing system optimization, improved protocols for handling, establishment of optimal environmental conditions and development of high-quality microdiets (Pinto et al., 2018). Taking this in consideration, the dietary supplementation of a commercial diet (CTRL) with curcumin and grape seed extracts stands out by even enhancing further the high growth observed in fish fed the CTRL diet.

The muscle growth is a highly controlled process with spatial-temporal expression patterns of MRF and other growth-related genes (Kitajima et al., 2018). The differences in the phenotype observed in the sole fed CC diet were accompanied by a concomitant and significant up-regulation of the *myod2* and the *mymk* transcripts. The *myod2* is a master regulator of the skeletal muscle for its visible effects in the recruitment of stem cells into the skeletal muscle lineage, as well as, in the proliferation and differentiation of myoblasts (Rudnicki and Jaenisch, 1995; Wardle, 2019). In turn, *mymk* is a recently identified gene that encodes muscle-specific proteins that directly govern the fusion process of myoblasts (Landemaine et al., 2014). We hypothesize that the up-regulation of both *myod2* and the *mymk* in fish fed CC diet could activate the differentiation and fusion of myoblasts that in turn would increase both the number and the proportion of large-sized fast-twitch muscle fibers observed in this study. Furthermore, these two genes were positively correlated with the largest sized fiber classes (>30 μm). Previous reports in pacu (*Piaractus mesopotamicus*) and sole larvae also suggest that an upregulation of *myod2* was related with an increase in hyperplasic growth (Leitão et al., 2011; Campos et al., 2013a). In rats, the administration of dietary curcumin also resulted in increased number of skeletal muscle fibers and was associated with increased expression of myogenic factors *myf5*, *myod* and *myog* (Chaudhary et al., 2019).

In addition to *myod2* and *mymk*, the inclusion of antioxidants also modified the expression of *mstn1* in postlarvae. In mammals, *mstn* gene is known to act as potent regulator of muscle growth, and a *mstn*-knockout fish highly increased muscle mass (de Santis et al., 2012; Kim J. et al., 2019; Kim J. H. et al., 2019). Contrarily to mammals, *mstn* transcription in fish species is ubiquitous expressed indicating an involvement in other physiological mechanisms as well as in skeletal muscle growth regulation (Campos et al., 2010; Li et al., 2012; Canada et al., 2016). Different stress situations that reduced fish growth were shown not to be correlated with the expression of *mstn* (Rescan, 2005). In transgenic lines of zebrafish (*Dario rerio*) and medaka (*Oryzias latipes*) overexpressing *mstn* prodomain (responsible for inhibition of myostatin function) showed an increase in the number of fibers, but no significative differences in fiber size or gross muscle mass (Xu et al., 2003; Sawatari et al., 2010). Furthermore, in rainbow trout (*Oncorhynchus mykiss*) primary myosatelite cells, the supplementation of myostatin in the culture medium stimulate the differentiation of this cells into myotubes by inducing the expression of *myf5*, *myod*, *myog* and *myhc* (Garikipati and Rodgers, 2012). Previous studies in sole also reported increased *mstn1* mRNAs levels in fast-growing groups of pre-metamorphic larvae and juveniles (Campos et al., 2013a; Canada et al., 2016). Therefore, *mstn1* seems to be associated with better growth performance in this species, but the mechanism behind this process needs to be clarified.

Muscle-specific ubiquitin ligases, *muscle atrophy F box/atrogin-1* and *muscle RING finger 1*, are critical regulators of myofibrillar protein degradation. Other studies showed that curcumin and grape seed extracts might decrease the expression of these genes and consequently decrease muscle protein degradation in mice (Wang et al., 2014; Ono et al., 2015). However, in the present study no changes in the expression of ubiquitin ligase *mafbx* and *murf1* were observed. Nevertheless, a positive correlation was observed between *mafbx* and the amount of very small fibers (< 5 μm). This correlation may be explained by the higher surface-to-volume ratio in small-sized muscle fibers which improves metabolic exchanges and consequently protein catabolism and turnover (Zimmerman and Lowery, 1999).

This study suggests that the supplemented extracts of curcumin and grape seed were able to promote growth in sole postlarvae preferentially by an up regulation of myogenic and muscle growth factors rather than a decrease in myofibrillar protein degradation. Growth is a highly demanding metabolic process, which involves a diversion of resources away from self-maintaining processes, such as redox system. The supplementation of curcumin and grape seed extracts has been well documented and improved oxidative status of several fish species. Therefore, the increase in growth and modulation of myogenic regulators observed in this work might be explained by a higher allocation of the energy budget to growth. A similar response in myogenesis modulation by upregulation of myogenic regulatory factors was observed in rainbow trout when fed supplemented diets with methionine and *in vitro* cells of rainbow trout and pacu supplemented with antioxidants (Villasante et al., 2016; Alami-Durante et al., 2018; Duran et al., 2019). Moreover, dietary inclusion of curcumin has been shown to upregulate the growth hormone gene expression and other growth factors involved in the regulation of myogenesis in tilapia (*Oreochromis mossambicus*) (Midhun et al., 2016).

A previous study in sole demonstrated methylation of the *myog* promoter is dependent on the thermal regime and is associated with changes in myogenic gene expression patterns (Campos et al., 2013b). There are also studies showing that curcumin, resveratrol and other dietary polyphenols can alter epigenetic characteristics by down regulating the *dnmt gene* expression (Pan et al., 2013; Frolinger et al., 2018). In the present study the expression of the *dnmts* did not vary significantly between dietary treatments. This lack of influence in the expression of *dnmts* by the experimental diets might be due to the late developmental stage considered (postlarvae). Previous studies reported changes in the expression of *dnmts* in earlier larval stages, such as pre or during metamorphosis, in response to environmental and dietary factors (Campos et al., 2013b; Firmino et al., 2017; Canada et al., 2018; Carballo et al., 2018). Periods of embryogenesis and early larval development might hence be the critical windows of highest sensitivity and metabolic plasticity, more prone to epigenetic changes. Nonetheless, a positive correlation was observed between expression of the different *dnmt* genes analyzed (*dnmt1* and *dnmt3bb.1*) and the proportion of large-sized fibers (>25 μm). Likewise, a positive correlation between *dnmt3a* and the mean fiber diameter was previously reported in sole feed different diets at mouth opening (2 DAH) suggesting a potential nutritional programming (Canada et al., 2018).

## Conclusion

The dietary supplementation of natural antioxidants extracts in Senegalese sole postlarvae diets (CC and GS diets) improved growth performance by increasing final weight and length. Postlarvae fed CC diet had the highest muscle CSA that resulted from a significant increase in the total number of fibers, and the highest proportion of large-sized fibers. The increased hyperplastic and hypertrophic muscle growth was associated with a concomitant and significant up-regulation of the *myod2* and the *mymk* transcripts in fish fed CC diet whereas the GS diet modified the expression of *mstn1* in sole postlarvae. The results in the present study provide new clues about the regulatory effects of the use of nutraceuticals on muscle growth and development in flatfish.

## Data Availability Statement

The raw data supporting the conclusions of this article will be made available by the authors, without undue reservation.

## Ethics Statement

This experiment was carried out by trained scientists and followed the European Directive 2010/63/EU of European Parliament and of the Council of European Union on the protection of animals used for scientific purposes and was approved by the Committee of Ethic and Animal Experimentation of the Centre of Marine Sciences of Algarve (CCMAR). The CCMAR (Faro, Portugal) facilities and their staff are certified to house and conduct experiments with live animals (‘group-1’ license by the ‘Direção Geral de Veterinaria’, Ministry of Agriculture, Rural Development and Fisheries of Portugal).

## Author Contributions

MX conducted the experiment, performed all the analytical analyses, analyzed all data, performed the statistical analysis, prepared the figures, and wrote the manuscript. SE, LV, and LC designed the study and supervised the research. MM and CC performed the analyses of RT-qPCR. RG and RC collaborate in experiment and sampling. VF contributed the histology analysis. LC formulated the diets. All the authors contributed to writing of the manuscript.

## Conflict of Interest

The two experimental diets (CC and GS) are included in the patent pending application PCT/IB2020/056001. The authors declare that the research was conducted in the absence of any commercial or financial relationships that could be construed as a potential conflict of interest. The reviewer MH declared a shared affiliation, with no collaboration, with two of the authors, CC and MM, to the handling editor at the time of review.
